# Conspiratorial misogyny and the radicalization continuum: male supremacy as a dominant ideology in contemporary extremism

**DOI:** 10.3389/fpsyt.2026.1893934

**Published:** 2026-08-28

**Authors:** Victoria Gurevich, Fielding Montgomery

**Affiliations:** Polarization & Extremism Research & Innovation Lab (PERIL), American University, Washington, DC, United States

**Keywords:** conspiracy, gender-based violence, male supremacy, public health, radicalization

## Abstract

Male supremacist ideology is quickly becoming one of the most prevalent forms of contemporary extremism in the world. In many ways, it is similar to how other extremist movements work, and the radicalization of its supporters follows familiar pathways. In other ways, the rise of male supremacy is both an outcome of increasing radicalization and an underlying risk for gender-based violence (GBV). This work aims to highlight key understandings of male supremacy as it relates to the manosphere and GBV. It also highlights how radicalization and ideology interact, how that radicalization leads to GBV, how male supremacy fills this ideological aspect of radicalization, and how conspiratorial thinking underpins much of male supremacist ideology. From there, we offer some practical risk and protective factors for practitioners, framed in a public health-informed understanding of radicalization prevention.

## 1 Introduction

An Ipsos and King’s College London poll from early 2026 surveying 29 countries found that “31% of Gen Z men agree that a wife should always obey her husband” (1). Among baby boomers, agreement was only 13%. The discrepancy between younger and older generations was maintained across questions that asked about discrimination against men and support for equality, with Gen Z consistently expressing more disapproval of and a greater sense of loss as a result of women’s equality (1). Socially progressive attitudes do not typically ripen with age; in fact, historically and along nearly every political dimension, younger generations have consistently held more progressive views than their predecessors (2, 3). Attitudes with respect to civil rights, climate change, LGBTQ+ acceptance, and reproductive rights have all historically trended more open and accepting among younger cohorts. Yet, among Gen Z men, and to a lesser extent women, attitudes toward feminism and gender roles have challenged this trendline, with another poll finding that Gen Z boys and men are more likely than older baby boomers to believe that feminism has done more harm than good (4).^1^

The gender divide among Gen Z and their general attitudes toward and backlash against feminism have become a popular topic of analysis in the last several years, with dozens of studies, surveys, and articles examining the nature and drivers of gender polarization. On the whole, Gen Z men remain progressive: they generally favor gender equality (with 62% of respondents from the same poll reporting that achieving equality between men and women is personally important to them), oppose discrimination, and hold more egalitarian views than many previous generations on many social issues (1, 5, 6). But what has captured researchers’ and journalists’ attention is the rapidly growing vocal minority emerging out of what is known as the “manosphere.” First used online in 2009 on male-centered blogs and forums, the term “manosphere” today describes the loose, largely online ecosystem of communities, influencers and content creators organized around male identity and grievance (7, p. 639). The manosphere encompasses a wide range of content—self-help advice, fitness, financial advice, gaming, and dating strategy—but what has emerged as its characterizing feature is its throughline of male supremacy: an ideology of hate based in the supposed genetic superiority of cisgender men and their ability to control women, as well as trans and non-binary individuals (8). Ironically, however, male supremacy also often functions from a position of vulnerability: the belief that men are disadvantaged, discriminated against, and owed a restoration of their value and authority (7, 9). It is this ideological core that situates the manosphere within a broader and more urgent conversation about extremism.

The boundaries of the manosphere are themselves subject to scholarly debate. Whereas some researchers treat the manosphere as a relatively coherent ideological ecosystem united by shared antifeminist politics (7, 10), others caution against overstating its unity, arguing that while the communities share surface features—such as a focus on grievance and male identity—the groups diverge significantly in their politics, tactics and relationship to violence making the manosphere a loose aggregation of communities rather than a cohesive unit of analysis (9, 11). For this analysis, we will use the term to describe the broader ecosystem, recognizing that the ideological throughline of male supremacy operates as a tendency across its communities rather than a doctrine.

With that being said, male supremacist ideology is quickly becoming one of the most prevalent forms of contemporary extremism in the world (8, 12). Male supremacy is rooted in the belief that cisgender men are inherently superior to, and thus have a right to social and political control and domination over, women, trans individuals, and nonbinary people (8). This is the foundational belief of male supremacist ideology, but it is not the face of it that most people first encounter. In any extremist movement or worldview, overt doctrine is rarely the first point of entry and not what often brings people in (13, p. 2). Instead, people are often drawn in through more relatable, accessible content that effectively speaks to what they need—often community, identity, a sense of purpose, or an explanation for their personal challenges—before incrementally radicalizing over time (14, 15). The manosphere operates along this continuum, with entry points that are culturally mainstream, and endpoints that call for domination and violence.

While the manosphere exists primarily online, its influence extends well beyond the internet, with far-reaching implications that affect culture, politics and public safety. Figures who espouse male supremacist views are among some of the wealthiest and most recognizable people in the world (16, 17). Andrew Tate has been identified as “the globe’s most prolific and most followed social media producer of masculinity-related content” (18, pp. 459-460) and a defining personality of the modern manosphere (19). He has amassed a following of tens of millions with a platform that normalizes the subordination of women and models and espouses an aspirational masculine identity built on domination and contempt (18, 20). Doiciar & Creţan (21) further argue that the Tate Brothers “exemplify how class privilege[d] men leverage wealth and influence to perpetuate harmful gender norms” (p. 14). Likewise, Elon Musk, the wealthiest man on the planet, has also amplified male supremacist views by endorsing calls for a “Republic of high status males” and “high T alpha males”, and decrying the “woke mind virus” (17, 22). The impact of this ideology has been reflected in legislative and cultural responses. Australia passed a social media ban for youth under 16, citing exposure to misogynistic content as among the harms that youth encounter online (23). Popular culture has sought to illuminate its dangers for the public; in the Netflix show *Adolescence*, a young boy is radicalized through manosphere content, and *The Pitt*, in which a mass attack is feared to have been motivated by an explicit hatred of women. What’s more, not only does male supremacy stand alone as a dangerous ideology that motivates violence in its own right, violent misogyny has also been identified as a significant predictor of other types of mass violence, underscoring the urgency of treating it as a serious public safety concern (12, 24).

This article takes up that urgency. In this conceptual analysis, we examine the rise of male supremacist ideology by contextualizing it within studies of radicalization and gender-based violence (GBV). Critically, we use “ideology” in this paper not as a cause for radicalization (to explain why it happens), but rather as a map that shows us where male supremacy takes its adherents. In particular, we argue that the role of conspiracy is a throughline connecting male supremacy with other prevailing forms of radicalization and GBV frameworks. Recognizing conspiratorial misogyny as a structural feature of this movement is thus central to violence prevention efforts. To that end, we introduce the public health-informed approach to violence prevention for its ability to identify risk factors, protective factors, and intervention opportunities across the prevention spectrum.

## 2 Radicalization

The academic study of radicalization soared in the aftermath of September 11, 2001, as governments and researchers sought to understand how individuals come to adopt and act on violent extremist beliefs (25, 26). Many early frameworks made two central assumptions: that radicalization led to terrorism, and that ideology was the cause of radicalization. However, decades of subsequent research have challenged these early claims.

To start, terrorism or political violence is rarely the outcome of any one individual’s radicalization. Politically motivated violence remains exceedingly rare, as many more people adopt radical beliefs than those who choose to show their commitment and support to a group, movement, or idea through violence (27). Neumann (28) has called this tension—between treating violence or mere cognitive commitment as the criteria for radicalization—the “principal conceptual fault line” of radicalization studies and is largely unresolved to this day (p. 873). Some theories and frameworks have attempted to navigate this by distinguishing between cognitive and behavioral radicalization, or by focusing specifically on the conditions under which radicalized beliefs translate into violent conduct. Moghaddam’s (29) “staircase to terrorism” model, for instance, conceptualizes radicalization as a series of ascending floors, with the vast majority of individuals occupying the ground floor of generalized grievance and only a vanishingly small number reaching the top floor of violent action. Similarly, McCauley and Moskalenko’s (27) “two pyramids” model separates the pyramid of opinion from the pyramid of action, arguing that the two are related but distinct phenomena that require different explanatory frameworks. While separating action from belief in models is an important recognition, treating these as two distinct processes has important implications for prevention: if radicalization and violence are not the same phenomenon, then the conditions that produce extremist beliefs and the conditions that produce violent action may require different interventions.

The second assumption that has since been challenged is the role of ideology, as the field of radicalization studies has come to understand that ideology, while important, is rarely the root cause of radicalization. But before turning to how ideology’s role has been reconceived in the radicalization literature, let us first clarify what the term itself denotes. Across social scientific traditions, ideology has been defined as a coherent system of political beliefs (30), as a form of discourse that naturalizes existing relations of power (31), and as a set of affective attachments and identity commitments that are only loosely organized into explicit belief (32). If ideology is primarily a coherent belief system, then exposure to and persuasion by ideas are the mechanisms that warrant attention. If it is primarily a discourse or an affective structure, then the social conditions that make certain narratives resonant and the identity needs they fulfill are where the analytical focus belongs.

The radicalization literature has increasingly moved toward the latter understanding, not only as more has become understood about the process of radicalization itself, but because the concept of ‘ideology’ has also been reexamined. In the view of ideology as an affective structure and discourse, it becomes a vehicle to radicalization rather than a driver (25, 33). This is not to say that ideology as a belief system is unimportant. The content of what an extremist movement believes and what it promises its adherents tells us a great deal about the worldview it cultivates, the grievances it speaks to, and the solutions it will propose. Instead, research suggests that ideology is better understood as a lens through which pre-existing needs and vulnerabilities are interpreted, rather than as the force that creates those needs in the first place. By reconceptualizing the role of ideology in radicalization as an affective system, research across different extremist movements and groups has found that what often propels individuals toward extremism is a complex interaction of psychological, social, and structural factors: grievance formation, identity threat, moral disengagement, the search for significance and belonging, and the availability of movements that offer compelling answers to those needs (14, 15, 33). Radicalization is thus not simply about the seductive power of ideas, but about the interaction between those ideas and the vulnerabilities those ideas are designed to exploit. Prevailing models have accordingly shifted toward understanding the pathways through which individuals become embedded in extremist movements: through organized groups, charismatic leaders, and, increasingly, online spaces that facilitate ideological reinforcement and social bonding (28, 34). The emphasis has expanded from what people believe to also encompass why they come to believe it, and what needs that belief is meeting. With these two developments in mind, radicalization is generally understood as a process by which an individual comes to adopt an increasingly extremist set of beliefs which may or may not lead to the willingness to condone, support or use violence to further those beliefs and goals (35, 36).

The shift away from centering ideological beliefs in the radicalization process is especially relevant for understanding male supremacist radicalization. Specifically, although ideological beliefs are not the root cause of radicalization, that does not mean they fail to serve as a central explanatory concept for understanding male supremacy. The difference to note is in ideology’s use as a diagnostic tool of radicalization, rather than a cause. While ideology does not generate the needs and grievances that draw people toward extremism, it does tell us what a movement promises its adherents, what worldview they will come to inhabit, what targets it designates, and what forms of violence it will authorize or celebrate. In the case of male supremacy, the ideological content (and its conspiratorial structure) is not incidental to the harms it produces but is constitutive of them. We therefore use ideology not as an explanation for why people radicalize, but as a map of where radicalization takes them.

Rather than emerging primarily through formal organizations or coherent ideological doctrines, male supremacist ideology has taken shape through diffuse narratives about gender, power, and social change, which are often spread and consumed through algorithmic content feeds rather than through deliberate ideological recruitment (7, 9, 37). The ideology of male supremacy is also sustained through the manosphere, which is a decentralized ecosystem of independent figureheads, rather than bounded organizational structures, which makes it highly adaptive and opportunistic, able to evolve quickly to meet individual vulnerabilities and cultural moments. In this way, male supremacy is less concerned with recruiting members into a clear movement than with gradually immersing individuals in a worldview that is characterized by conspiracy and interprets personal grievance through gendered plots of victimization, offering male dominance and masculinity as the solution.

One aspect of radicalization which male supremacy has challenged is the role of the internet. Research into how the internet affects radicalization concluded early that the internet facilitates, rather than causes radicalization; acting more as an accelerant rather than an independent source of extremism (38). The digital environment has fundamentally transformed since this conclusion was reached. The rise of social media algorithms optimized for engagement, the proliferation of short-form video content, and the recent integration of artificial intelligence into content recommendations and generation have produced an internet that is categorically different in its capacity to shape belief and behavior today than when it was first studied. The consequence of these changes has been the shift away from the intentionality that individuals used to be required to apply when navigating the internet, to it now being an environment that actively curates and escalates content toward more extreme positions (39). Male supremacist content which spreads through platforms that reward provocation and engagement has been the primary beneficiary of these shifts.

The radicalization literature offers a powerful lens through which to examine male supremacy: it decenters ideology as the causal factor, recognizes grievances, identity threats and explanations as the vulnerabilities that the worldview exploits, and speaks to the worldview that adherents may come to adopt. While radicalization is a suitable lens through which to examine male supremacy, it must be studied alongside a second body of scholarship: gender-based violence.

## 3 Gender-based violence

Gender-based violence (GBV) refers to harmful acts directed at individuals on the basis of their gender, and encompasses a wide range of behaviors including stalking, harassment, sexual assault, intimate partner violence, sex trafficking, and femicide, among others (40). Although GBV affects people of all genders, it is structurally gendered in its distribution: women and girls are disproportionately its targets, and men are disproportionately its perpetrators (41). This pattern holds across cultures, geographies, and socioeconomic contexts (42). The field that has developed to study, document, and respond to these patterns is among the most empirically rich in the social sciences and is critical to addressing male supremacy.

The feminist movements of the 1960s and 70s can be credited with professionalizing the GBV field due to their advocacy for naming violence against women as a social problem rather than a private, family matter. The foundational claim of the field is thus that violence against women is not random or interpersonal in origin but structural and rooted in patriarchal systems that normalize and enforce male dominance and female subordination (43–45). To this end, Kelly’s (46) concept of the “continuum of sexual violence” explains how gendered harm operates along a spectrum. Specifically, it argues that sexual violence should not be understood as discrete or exceptional acts, but as a range of behaviors connected by a common logic of male entitlement and female objectification. In the four decades since its development, the continuum framework has been extended and refined yet remains central to how GBV is understood and how male supremacist ideology operates, with misogynistic humor and online harassment on one end, and rape and mass violence on the other.

Despite the tremendous depth and extensive knowledge of the GBV field, it has been understudied as a form of radicalization. There are frameworks and accounts for how violent misogyny is learned, normalized, and perpetrated, but the field has not consistently engaged with the radicalization literature to consider how individuals come to adopt and act on extremist beliefs. For its part, the radicalization field has also not consistently incorporated insights from GBV either, instead treating GBV as a downstream symptom of other forms of extremism rather than as an organizing ideology in its own right. The gap we are left with is that two of the most relevant bodies of scholarship for understanding male supremacy have largely developed in isolation from one another.

This article offers to bridge that gap. Male supremacy is at the same time a process of radicalization and a driver of GBV. As male supremacy’s cultural reach expands and rates of violence against women rise, integrating insights from radicalization and GBV is becoming increasingly urgent in order to identify, interrupt and prevent its harms.

## 4 Male supremacy as ideology

In order to understand the pipeline for radicalization toward GBV, one needs to grapple with the ideology of male supremacy that underpins much of not just GBV and misogyny, but even broader extremism. As a system, male supremacy is defined as a “cultural, political, economic, and social system in which cisgender men disproportionately control status, power, and resources, and women, trans men, and nonbinary people are subordinated (p. vii)” (47). However, as Rothermel and Kelly (48) note, “Feminists have argued that male supremacy is both a central ideological element driving violent terrorist attacks and a broader system of unequal gender relations expressed in ideas about male entitlement, misogyny, and anti-feminism” (p. 544). Thus, the authors argue that one can be radicalized into “male supremac*ism*,” or the radical ideological component that reestablishes and upholds male supremacy as a system (48, p. 544). While Rothermel and Kelly (48) have incorporated the views of male supremacy as a systemic condition and a radical ideological commitment into one definition, it is worth noting how the distinctions between these two views have shaped and been shaped across disciplines. Within feminist theory, the view of male supremacy as a structural arrangement of a patriarchal society is an organizing logic of most societies and not an extremist fringe position (43, 49). Extremism literature, by contrast, uses the term to describe a conscious, explicit belief in the inherent superiority of men and the rightful subordination of women that is distinct from patriarchal norms, even if it is continuous with them (8, 12). The differences in these two traditions have implications for where change efforts should be directed: frameworks that locate male supremacy only at the extremist fringe risk missing the broader cultural milieu that normalizes and sustains it, whereas frameworks that locate it everywhere lose precision about what distinguishes radicalization from socialization. This paper works across both registers, treating male supremacy both as a structural condition that shapes cultural norms and an ideological formation that can motivate violence.

With that in mind, male supremacy as an ideology has gained a foothold in a number of online communities. This diffuse group of online reactionary masculinities and misogynistic discourse can broadly be understood as comprising the “manosphere” (10, p. 1926). Given the diffuse nature of the manosphere, there are a number of different ways to understand its subdivisions. As Han and Yin (10) argue, the manosphere can fall into two main categories—antifeminist discourse and masculinist narratives (p. 1926). In both of these subcategories, male supremacy enacts itself by either excluding women or policing what it means to be an ideal man. Another useful understanding of the manosphere is the subgroups that loosely comprise it. As Ging (7) suggests, there are five key interest groups of the online manosphere: men’s rights advocates (MRAs), men going their own way (MGTOW), pick up artists (PUAs), traditional Christian conservatives (TradCons), and gamer/geek culture (p. 644). From our own research, we would also add online incel (involuntary celibate) communities and some elements of the femosphere, such as tradwives, who work to maintain male supremacy from a woman’s perspective (50–52).

Regardless of how one subdivides the ideological underpinnings of male supremacy, one unifying component of this ideology is the “red pill.” As Ging (7) argues, “Central to the politics of the manosphere is the concept of the Red Pill, an analogy which derives from the 1999 film *The Matrix …* The Red Pill philosophy purports to awaken men to feminism’s misandry and brainwashing, and is the key concept that unites all of these communities” (p. 640). Indeed, it is this red pill portion of male supremacy ideology where radicalization and threats of harm and violence begin to come into play. Taking the red pill acts as an acceptance of extreme grievance, where men feel that they are being oppressed by women and society generally. As Miller-Idriss (53) notes, “In case after case, misogynistic rage against women, the LGBTQ+ community, or prior reports of intimate partner violence are a documented part of [mass violence] perpetrators’ histories, even when their official motives lie elsewhere” (p. 6). Thus, left unchecked, male supremacist grievance is a possible vector toward interpersonal and mass violence.

However, while the connections between male supremacy, grievance, and violence are clear and prevalent, they are often understudied or not understood in connection to radicalization. Indeed, as Bosman et al. (54) highlight across many profiles of mass shooters, that “one common thread that connects many of them—other than access to powerful firearms—is a history of hating women, assaulting wives, girlfriends and female family members, or sharing misogynistic views online.” Obviously mass violence is one of the worst outcomes of gendered grievance, but this radicalization also leads to sexual violence, domestic abuse, hate crimes, and self-harm. The potential spread of content promoting these grievance ideologies is alarming too. A report from the UN notes that between 40 and 50 percent of young men and adult men in the United States trust one or more manosphere influencers, 69 percent of men and 72 percent of women are concerned about the sexist rhetoric they see online, 58 percent of girls and young women have experienced online harassment, and that such spread provides possible rabbit holes for further radicalization (55). A report on digital male supremacy from the Polarization & Extremism Research & Innovation Lab (PERIL) and Over Zero (56) found that on fringe platforms such as 4chan, Gab, Telegram, and Truth Social, where radicalization potentials are heightened, as many as 15% of all posts included male supremacist content. Even further, for every 1,000 words posted on these websites, as many as 2.5 are overtly male supremacist, even when including common stop words like and, the, or, etc. As such, the problem of male supremacist radicalization is both pervasive and highly consequential.

One element to note is the role of age. The prevalence of male supremacy among adolescent boys complicates the prevailing views of radicalization in an important way. Youth as young as twelve are being drawn into male supremacist content and communities; this is a demographic that has not yet experienced romantic rejection, job loss, or social marginalization that radicalization research typically identifies as grievance pathways. As such, for adolescents this young, it is difficult to argue that ideology speaks to lived experience in any direct sense. Whereas the field of radicalization has broadly decentered ideas as being the driving force of radicalization, instead emphasizing needs and grievances that come from lived experiences, with male supremacist radicalization trending younger and younger, lived experiences seem to be missing in terms of what’s resonating, leaving the ideas themselves. Young boys are encountering and believing the claims of male supremacy on their own terms rather than as a framework for interpreting personal experience. More research is needed before firm conclusions can be drawn, and the child development field in particular must be brought into the conversation. Possibly, adolescent engagement with male supremacy may be less about genuine belief in the subordination of women and more about identity formation, parasocial attachment, and the emulation of figures who project confidence, status, and success regardless of the content of what those figures actually say. In other words, young boys may be following manosphere influencers for the same reasons they follow any aspirational figure: not because they have adopted their worldview, but because they model a version of masculinity that feels compelling. Whether the pathway runs through ideology, personal identity, or admiration, the trend of youth radicalization into male supremacy demands serious attention and suggests that radicalization frameworks may need to account for developmental stages as a meaningful variable.

In any case, understanding male supremacist radicalization through a public health-informed approach, including a recognition of risk and protective factors, is essential. However, first it is important to understand one key narrative and psychological element of male supremacist radicalization: conspiratorial thinking.

## 5 Male supremacy and conspiratorial mindsets

While male supremacy contains a wide array of degrees and intersecting forms of hate, one of the more unifying concepts we have identified across our research is a conspiratorial style of online rhetoric (57–62). In many ways, this is connected to the concept of the red pill outlined above. The red pill itself is a deeply conspiratorial mindset, which suggests that women, feminism, and society as a whole are conspiring against men. We will discuss further how such a conspiratorial mindset is embedded within male supremacy ideology, but this understanding also helps to explain how male supremacy intersects with other related forms of hate like white supremacy, antisemitism, homophobia, and the like.

Although the concept of conspiracy theory has a popular understanding, it is worth problematizing and clarifying its foundations. Hofstadter’s (63) foundational account of the “paranoid style” of conspiratorial thinking framed it as a psychological disposition characterized by grandiosity, persecution, and apocalypticism. Although popular perceptions may have maintained this characterization, this view has been subjected to significant revisions in the literature. The primary criticism argues that Hofstadter’s framework pathologizes political dissent, conflates the rhetoric of marginalized movements with clinical paranoia, and obscures the ways in which conspiratorial reasoning can be a rational response to genuine experiences of institutional betrayal or exclusion (64, 65). Moreover, it has been noted that institutions do in fact conspire, and that the line between a conspiracy theory and a well-founded account of institutional wrongdoing is often drawn retrospectively and politically (66). With this in mind, recent scholarship has differentiated between conspiracy theories and a “conspiracy mentality,” or disposition toward conspiratorial explanations as a general approach to understanding the world (67, 68). It is in this latter sense as a generalized epistemic orientation that we use the term throughout this analysis.

Indeed, there are a few key concerns related to how a conspiracy mentality functions in the meaning-making processes of male supremacist conspiracy theorists. First, as Rice (69) indicates, conspiracy is “a pretender to true evidentiary processes, a sham passing off narrative as true evidence” (p. 5). Time and time again, we have noted this process at play in the posts of misogynistic users and influencers we have observed, where the narrative of gendered grievance and hate overrides rational evidentiary processes and discussion. For example, multiple anonymized posters in our own research, when presented with factual refutations of their positions, have responded with claims that such evidence is “feminist/transgender psyop propaganda” or other such refutations. This is a prime example of how narrative overwhelms evidence: anything in support of one’s position is, as another anonymized user put it, a “fact-checked, reputable source, you … cultist.” And anything that goes against the male supremacist grievance narrative is a conspiratorial psyop. Such rejection of established evidentiary processes is one possible risk factor for conspiratorial misogyny that future research could examine.

While conspiracy can and does often reside at the level of evidentiary processes and hateful narratives, it can go deeper. For example, Hameleers (70) notes three facets of conspiracy reasoning: elements of reality being “attributed to hidden, goal-directed, and evil forces,” the interpretation of sociopolitical events as ultimate battles of “good versus evil,” and the suggestion that there are hidden truths covered up by mainstream knowledge bases (p. 40). A strong example of this combination of conspiracy into hate are black pill incels (involuntary celibates), who believe that there are hidden forces keeping them down and promoting “Chads and Stacys,” with gender-based mass killings promoted as a battle of good versus evil, and hidden truths being covered up or ignored by “normies,” or mainstream society. Indeed, as another anonymized poster on an incels forum claimed, normies and Chads will “always find a reason to justify their dislike of you.” The user further warns, “God forbid you somehow come into a position of power. I remember the guy who went ER in a Walmart a few years ago did so because … his employees bullied and mocked him.” Very important to note here that going “ER,” in incel terminology, means going “Elliot Rodger,” an infamous incel who committed mass violence. Thus, this highlights how there is a supposedly hidden battle between women/normies/Chads, and incels, that society is inherently evil, and that such truths are covered up by the very “normies” who comprise mainstream society.

Such understandings of the conspiratorial mindset can similarly be applied to red pill adherents. Indeed, as Van Valkenburgh (71) notes, in order to be a “red piller,” one “must recognize the existence of the feminist matrix before developing the skills to find freedom from its influence” (p. 89). The very pop-culture reference at the heart of this mindset, *The Matrix*, establishes this conspiratorial mindset through its narrative that suggests the world is a simulation and that one needs to see through the illusion and wake up to reality. That conspiratorial narrative is then mapped onto the red pill, where the “matrix” is feminism. Thus, given that nearly all corners of the manosphere adhere to the red pill to a certain degree, the conspiratorial mindset related to the red pill infiltrates the majority of male supremacist ideology.

Finally, another unifying aspect of male supremacist and related ideologies of hate as it relates to a conspiratorial mindset is what Hofstadter (63) terms the “paranoid style” in his foundational work. This psychological and rhetorical styling suggests “a vast and sinister conspiracy” bent on destroying “a way of life,” with supposedly confirmatory events and experiences comprising a conspiratorial “*motive force* in historical events” (p. 29). Those who fall into the paranoid style begin not just espousing targeted hate at one group, like women, but rather become anti any number of groups that they view as a part of such vast conspiracies. This explains the consistent findings of our research that hate is not isolated, but almost always intersects (56, 72). It could be easy to dismiss such all-encompassing hateful mindsets as irrational, but as Neville-Shepard (73) cautions in his revision of the paranoid style, there is an “anti-establishment” and “broad-based political ideology” at play that explains the logical appeals of this way of thinking (p. 129). As such, it is essential to “continue grappling with the consequences of mediated disinformation and racialized misogyny” (74, p. 333).

Antisemitism is another deeply conspiratorial form of hate that often overlaps with male supremacy ideology for those who engage in a paranoid style of rhetoric. One demonstration of this overlap comes from an anonymized 4chan poster arguing against age of consent laws, suggesting that only unintelligent normies believe “the Jewish lie that sexually mature young ladies are somehow undesirable and/or forbidden to want.” Thus, the male supremacist and pedophilic narrative of objectifying underage girls based on their supposed sexual maturity is combined with an assertion that age of consent laws are a Jewish conspiracy. This is then often linked into the Great Replacement conspiracy, which is “rooted in the belief that the white race is under threat of extinction at the hands of Jews and other minorities” (75). Thus, one can see how these conspiracies related to the hidden, “evil” Jews allow for a perceived battle of good versus evil, covered up by the mainstream, where men’s intimacy grievances are targeted at women and Jewish people alike.

This co-habitance of antisemitism and male supremacy is fairly prevalent too, as a study by PERIL and Over Zero (56) found that on fringe platforms, as many as 4% of posts overtly engage simultaneously in both antisemitism and male supremacy. This type of conspiratorial thinking is then very frequently cross applied to other forms of racist, homophobic, and ableist hate. For example, Cioran et al. (76) demonstrate how male supremacy and gender violence have been targeted at Roma women both in Romania and Spain. The rhetorical and psychological movement toward the paranoid style that encompasses some of such racism and male supremacy might point toward another potential risk factor that unifies the different realms of misogyny and hate observed in our research. Thus, while trying to counteract misogyny and other forms of hate based on content proves difficult at times, further research on if conspiratorial and paranoid mindsets comprise behavioral risk factors for GBV may prove useful when considered in the context of a public health approach to radicalization.

## 6 Discussion

The public health approach treats extremism as a negative health outcome (similar to any public health problem, from cancer to car accidents). And if extremism is viewed as a health outcome, then it can also be treated and, ideally, prevented. This orientation represents a meaningful shift as extremism has traditionally been framed as a problem of criminality, to be addressed through securitized measures that focus on suppression, surveillance, and elimination, rather than recovery and prevention. This framing has not only shaped policy responses but has also influenced the questions that researchers ask: whereas criminal frameworks ask who to punish and how, public health asks what conditions produce harm and how those conditions can be changed.

The public health approach emerged as the dominant framework for addressing injury and disease in the mid-twentieth century and informed population-level health interventions such as seatbelt legislation, smoking cessation campaigns, and childhood vaccine programs (77). The public health approach’s application to violence prevention came later and gradually, with a 1993 report from members of the CDC that established a four-step public health approach to violence prevention that remains the prevailing framework to this day (78). The violence that this framework sought to address was, however, largely interpersonal and situational—assault, homicide, suicide, and community violence—and was not focused on “extremist” violence that people engage in because of radicalization (a process which was understood at the time to be predicated on ideology). Ideology did not map onto public health logic; beliefs were understood as personal decisions, not exposures or risk factors amenable to population-level intervention. However, as the role of ideology in radicalization was refined (recognizing that ideology was not the cause of violence, but a vehicle for how psychological and social needs could be met), extremist violence became legible for the public health framework. This made way for extremist violence to be incorporated into public health violence prevention frameworks and upstream interventions to be seen as possible (79, 80).

An important note on terminology. While the public health approach is an established, systemized, and empirically validated framework, its adoption within extremism fields is best described as public health-informed rather than public health proper. The key distinction lies in the institutional context in which the work takes place, who supports the work, and the resources available to it. The formal public health approach operates within highly regulated systems, health departments, epidemiological surveillance networks, state and national agencies, hospitals, etc. As such, individuals are highly trained in order to work with standardized clinical protocols and oversight structures. The public health-informed approach to extremism, on the other hand, works in community, educational, and policy settings that are not connected by any standardization of protocols, oversight, or training. While the conceptual tools of the public health approach can translate into these settings, implementation looks quite different. Community organizations, educators, threat assessment professionals, and mental health providers are asked to do what they can. The result is a field that is characterized by promising approaches yet significant gaps, where the quality and availability of interventions depend on local capacity, institutional will, and the know-how and buy-in of individuals rather than any standardized system that is designed to ensure consistent care.

Aside from the differences in implementation, the public health approach offers an architecture for how to think about intervention. Beginning with primary prevention, this level targets the general population before any radicalization has occurred; it focuses on building resilience and reducing vulnerability. In traditional public health programs, these are the interventions that focus on the general population regardless of individual-level behaviors, such as seatbelt laws, healthy lunch programs, and anti-smoking campaigns: they offer benefit broadly. Applied to extremism prevention, these are programs that teach media literacy, build critical thinking, and affirm individuals’ sense of belonging, purpose, and positive self-identity. And extending this to male supremacist radicalization specifically, primary prevention efforts can be those that strive to reshape the cultural norms around masculinity that male supremacy exploits by targeting rigid, hierarchical constructions of manhood that equate worth with dominance and contempt (81, 82). In particular, studies that consider the “man box” mentality have found that men who strongly endorse restrictive male stereotypes are significantly more likely to perpetrate violence and be victims of violence themselves (83).

Secondary prevention engages individuals and communities at elevated risk; in public health, this can include screenings and tests for individuals in higher risk groups, such as the recommendations for colonoscopies for men over 45 or mammograms for women 40 and older, or how campaigns or advertisements for behaviors such as drinking and driving or gambling may be targeted to specific venues or events where that risk is elevated. For extremism prevention, programs such as Behavioral Threat Assessment and Monitoring (BTAM) are becoming increasingly prevalent in communities and school districts across the country for offering a structured assessment and intervention framework to address vulnerabilities and risk factors before they escalate.

Finally, tertiary prevention focuses on those already affected by disease or injury, prioritizing recovery and rehabilitation (such as chemotherapy for cancer treatment or physical therapy following injury). Applied to extremism, these are desistance and exit programs for individuals already radicalized, focusing on getting them to leave their groups or movements and addressing the underlying needs that brought them in. Exit and disengagement programs for male supremacist radicalization remain underdeveloped relative to those for other extremist movements, but emerging work suggests that addressing the underlying relational and emotional needs, rather than focusing on ideological deconstruction alone, produces better outcomes (48).

### 6.1 Risk and protective factors

Building on the public health-informed model of addressing radicalization, especially as it relates to conspiratorial misogyny, it’s important to consider risk and protective factors that can impact an individual or cohort’s likelihood of radicalization. We understand risk factors as characteristics or conditions that increase the likelihood of radicalizing and/or committing extremist violence. Protective factors, then, are characteristics or conditions that decrease the likelihood of radicalizing and/or committing extremist violence. Testing and recognizing risk and protective factors and then working to minimize the former and expand the latter is a crucial aspect of using the public health model to address conspiratorial misogyny. Critically, risk factors and protective factors are not predictive: their presence or absence in an individual’s life does not determine outcome. No single risk factor causes radicalization, and no single protective factor prevents it; rather it is the accumulation and interaction of multiple factors across individual, relational, community and societal levels that shapes vulnerability and resilience.

In terms of conspiratorial mindsets, this work has already identified a few potential risk and protective factors that future research should further attempt to disprove or confirm. First is a willingness to reject evidence and reasoning that conflicts with one’s own viewpoints. Indeed, a response to this possible risk factor would be fostering critical thinking about sourcing and manipulation techniques being used by posters. A feeling of abandonment or “us vs. them” against general society is another possible risk factor. Pro-social protective factors could be a useful response here.

Further, research on specific populations as they relate to conspiratorial mindsets is useful. For example, Byrne et al. (84) conducted a systematic review of conspiracy theory beliefs in adolescents. One finding for them is that “it might be possible that for adolescents whose cognitive development is delayed, affected by dysfunction, or for those with lower cognitive ability, conspiracy theory beliefs are more likely to develop during this time” (p. 933). Further, the authors argue that “subclinical pathological factors, such as schizotypy and delusional thinking could explain the relationship between age and belief in conspiracy theories” (84, p. 933). Dyrendal et al. (68) found that for conspiracy theory belief, “the main path runs from schizotypal thinking via paranormal beliefs and especially conspiracy mentality to belief in specific conspiracy theories” (p. 5). As such, while these subclinical factors function as potential risk factors, possible protective factors could include better diagnosing and treatment of adolescents displaying signs of such cognitive factors. Indeed, for adolescent patients displaying particularly strong signs of conspiratorial thinking, proper testing could help uncover and prevent underlying factors before further radicalization occurs.

In terms of the “paranoid style” discussed above, such paranoid thinking may be related to adverse childhood experiences, including neglect and abuse (84–86). This could ultimately be related to the finding of Martinez et al. (87) that “a bias towards mistrust is associated with greater paranoia” (p. 391). Indeed, a history of violent victimization, an established CDC risk factor for intimate partner violence and teen dating violence, might very well lead to a paranoid style of thinking, in which hateful, anti-establishment views fester within an individual or among a group (88). Thus, considering possible paranoid mindsets when dealing with patients with histories of neglect, abuse, or general mistrust may be a useful prevention step. Relatedly, anxiety seems to play a role in conspiratorial thinking (84, 89). As such, recognition of conspiratorial mindsets when dealing with anxiety patients, as well as more general proper diagnosis and treatment of anxiety, may be important for treating and preventing such conspiratorial thinking, as psychological/mental health problems are another well-established risk factor for intimate partner violence and teen dating violence (88).

While we have identified conspiratorial thinking as one of the key psychological underpinnings of misogynistic belief systems, it’s important to recognize the impact of such misogyny as it develops. Indeed, a survey study conducted by Rottweiler et al. (90) found that misogyny itself “predicts violent extremist intentions, willingness to engage in interpersonal violence and increased support for violence against women via revenge planning and hypermasculinity” (p. 287). This finding is consistent with a substantial body of GBV research documenting that attitudes of male entitlement and hostility toward women are among the strongest individual-level predictors of intimate partner violence and sexual assault perpetration (81, 82). What the radicalization literature adds to this picture is the mechanism that misogynistic attitudes, when embedded in a conspiratorial worldview that designates women as an enemy class, may not be merely correlated with violence but actively cultivated toward it. Thus, misogynistic beliefs might very well function as a risk factor for violence against women. This violence against women impacts an estimated “10-53% of women worldwide” and “is strongly associated with physical, mental, emotional, sexual, and reproductive health problems and death” (91, p. 211). As such, future research and intervention work can and should further test and address these issues at multiple levels of potential risk factors: at the level of conspiratorial thinking, at the level of misogynistic beliefs, and at the level of prevention of GBV. This could then be incorporated with existing GBV prevention recommendations, such as Sánchez et al.’s (92) call to continue producing evidence, improving the practice of health service professionals, building formal and informal support networks, fostering programs based in dialogue and equitable relationships, and developing programs aimed at empowerment and financial autonomy of groups prone to experiencing GBV (p. 13).

### 6.2 Challenges

Addressing male supremacist radicalization through a public health-informed framework is not without significant challenges, many of which reflect broader, structural conditions in society. Some of the most well-established drivers of radicalization are distrust in society and institutions, social isolation, and loneliness (14, 79, 93), which are by most measures at a historic high (94). Political polarization in the United States has eroded public confidence in government, science, media and civil society, creating an environment that is rife for conspiracy-laden narratives to emerge, like manosphere content and its conspiratorial male supremacy ideology.

The epidemic of male loneliness also compounds the challenges of prevention. Research has consistently documented rising rates of social isolation among men in particular, with men reporting fewer friendships, less social support, and greater disconnection than at any previously recorded point (95). The masculinity literature offers important context here: research demonstrates that adherence to traditional masculine norms—such as emotional stoicism and self-reliance—is a significant predictor of social isolation, resistance to help-seeking, and poor mental health outcomes among men (96, 97). And loneliness is a significant vulnerability for radicalization as the needs for social belonging and connection are met by harmful groups and movements. The manosphere exploits this need for connection by offering community and an opportunity to feel seen, valued, and justified. As such, a public health-informed approach to dealing with male supremacy and conspiratorial misogyny is needed now more than ever.

## Footnotes

1 It is important to note that while the data on gender attitudes is consistent across multiple surveys, interpreting the data is not straightforward. What the data most reliably shows is that male identity and feminism have become increasingly antagonistic reference points for a significant subset of young men. What is not known is how stable or deep-seated the ideological commitment backing those responses is. In one view, holding anti-feminist beliefs may signal in-group affiliation with manosphere culture rather than attitudes about gender equality itself.
